# Anemia Awareness Program in a Suburban Middle School: A Pilot Study Promoting Students as Public Health Advocates in Their Community

**DOI:** 10.7759/cureus.59238

**Published:** 2024-04-28

**Authors:** Indu Saxena, Manoj Kumar, Ram Diwakar, Alka Shukla, Anil K Yadav, Amar P Kaur, Navneet Ateriya, Ashish Arvind

**Affiliations:** 1 Biochemistry, All India Institute of Medical Sciences Gorakhpur, Gorakhpur, IND; 2 Physiology, Maharshi Vashishtha Autonomous State Medical College, Basti, Basti, IND; 3 General Medicine, Maharshi Vashishtha Autonomous State Medical College, Basti, Basti, IND; 4 Pediatrics, Maharshi Vashishtha Autonomous State Medical College, Basti, Basti, IND; 5 Dentistry, Maharshi Vashishtha Autonomous State Medical College, Basti, Basti, IND; 6 Forensic Medicine and Toxicology, All India Institute of Medical Sciences Gorakhpur, Gorakhpur, IND; 7 Physiology, All India Institute of Medical Sciences Gorakhpur, Gorakhpur, IND

**Keywords:** rural area, a pilot study, middle school students, health education & awareness, iron deficiency anemia (ida)

## Abstract

Introduction: Almost a quarter of the people on earth are anemic, and most of them reside in regions of sub-Saharan Africa and South Asia. Anemia in children is linked with impaired cognitive and motor development and affects the future earning capacity. The most common cause of anemia is iron deficiency. The Indian Government has initiated multiple programs for the eradication of anemia. The prevalence of anemia has not decreased despite the improvements in the country’s economy. It increased from 58.7% in 2015-16 to 67.1% in 2019-21 in children and from 50.4% in 2015-16 to 52.2% in 2019-21 in pregnant women. Maternal education, socioeconomic status, and number of children in the family are some factors that influence the prevalence of anemia. As these factors cannot be improved in a short time, we aimed to increase awareness about this issue by targeting school students from rural/semi-urban backgrounds.

Methods: This pilot study aimed at promoting school students as public health advocates in their community. Anemia Awareness Program was conducted in a local middle school in the suburban area, which was attended by 153 class eight students (72 female). Pre- and post-test questionnaires comprising 20 multiple-choice/true-false type questions were used. Pre- and post-test scores were obtained. The second part of the study was the identification of students with anemia. Blood hemoglobin levels of 127 students (58 female) were measured from venous blood samples. The students were also asked to inform their friends/relatives about anemia and to send people with symptoms of anemia to the free two-day Anemia Awareness Camp organized by the Medical College Hospital for check-ups.

Results: The mean post-test score (15.68/20) was much higher than the pretest score (2.99/20). Thirty-eight (25 female) out of 127 students had mild/moderate microcytic hypochromic anemia, suggesting iron deficiency. Thirty-two persons visited the free health camp to receive information from the students, of whom four had normal hemoglobin levels.

Conclusion: This pilot study showed that physician-conducted anemia awareness programs are relatively low-cost methods to spread information among the general population in India.

## Introduction

Anemia, a condition in which the oxygen-carrying capacity of blood is reduced, is one of the major public issues in developing countries. The global prevalence of anemia across all ages was 24.3% (95% uncertainty interval 23.9-24.7) in 2021 [1]. This corresponds to 1.92 billion prevalent cases, most of them in countries of sub-Saharan Africa and South Asia.

In pregnant women, anemia increases the risk of preterm labor, short gestation, low birth weight, stillbirth, perinatal and postnatal mortality, infections, postpartum hemorrhage, etc. [2]. In preschool children, anemia is associated with increased morbidity and mortality [3,4]. Impaired cognitive and motor development, low performance in school, tiredness, and fatigue in school-age children have been linked with anemia [5,6]. The symptoms of anemia are diverse in adults and include fatigue, light-headedness, headache, pallor, tachycardia, palpitations, chest pain, dyspnea, cold distal extremities, claudication, and difficulty in concentration [7]. In older adults (more than 65 years of age), anemia is a risk factor for hospitalization and increased all-cause mortality [8] and can be due to nutritional deficiencies, chronic kidney disease, chronic inflammation, and occult blood loss.

Nutrient deficiency, especially iron, is the most common cause of anemia [9]. Other nutrient deficiencies include deficiency of proteins, vitamins (B12, folic acid, pyridoxine, and ascorbic acid), and copper. Besides nutritional deficiencies, anemia can be due to acute and chronic infections, intestinal parasites, bleeding disorders, inherited conditions, etc. According to the Global Nutrition Survey conducted in 2016, India ranked 170 out of 180 countries for anemia among women. Anemia is present in more than 40% of adolescent girls and menstruating adult women [10].

Anemia lowers productivity in adults and causes a loss of up to 4% of gross domestic product [11]. The lack of anemia reduction is surprising as India has been showing remarkable economic growth [12] since anemia rates decline approximately a quarter as fast as income increases [13]. Treatment and prevention of iron deficiency anemia (IDA) are easy, yet the incidence is not decreasing at a satisfactory rate [14].

Factors driving the reduction of the incidence of anemia include nutrition and health interventions, improvements in maternal schooling, improvement in sanitation, fewer young children per household, and socioeconomic status of the family [15]. The socioeconomic status of the family and educational levels cannot be improved in a day; therefore, we have aimed to increase awareness about these issues, targeting the most accessible population group, the school children. This pilot study was aimed at providing information related to anemia to school students and observing its effect.

## Materials and methods

This pilot study for training students to become health advocates in their community was conducted on students enrolled in the eighth standard of a middle school operating in a suburban area, after obtaining permission from the school principal and from the Institute’s Ethical Committee. A total of 164 (74 female) students were enrolled in three sections of class eight. The informed consent forms were distributed in classes two days before the program to obtain the signature of the student’s parent. Details of participants are summarized in Table 1.

**Table 1:** Details of students participating in the Anemia Awareness Program

Class VIII	Section A	Section B	Section C	Total
Female students enrolled	25	24	25	74
Female students present on the day of the program	24	24	24	72
Male students enrolled	30	32	28	90
Male students present on the day of the program	27	29	25	81
Students attending the program and participating in pre- and post-tests	51	53	49	153
Female students with signed informed consent for Hb test	20	19	19	58
Male students with signed informed consent for Hb test	22	27	20	69

Anemia awareness in students

The Anemia Awareness Program was conducted during school hours (8 am to 1.20 pm). The morning session started at 8.00 am with a pretest and included interactive discussions on what is anemia, the symptoms and causes of anemia, IDA in children and women, the effect of anemia (on patient, family, and country), and the role of healthy diet and hygiene. Each session of 40 minutes was followed by a 10-minute break for activities or snacks. Post-test was conducted after the educational activity was complete. Blood samples were collected from students whose parents had signed the informed consent form (a total of 127; 58 females) by four teams of physicians and technicians. The samples were sent to the Diagnostic Lab of the Medical College for analysis on a three-part automated hematology analyzer from Medonic.

Awareness in society

The students were asked to share the information with their families and friends, with the idea of "each one, teach at least one." They were also asked to send their family members or friends with suspected anemia to the Medical College Hospital (MCH) for free-of-charge blood hemoglobin measurement and routine check-ups (the MCH had organized a two-day free camp for anemia awareness). Photocopies of patient admission forms were to be submitted to the school office by the student concerned within seven days and were collected by our team at the end of seven days.

Data analysis

T-test was used to compare scores of pre- and post-tests between female and male students. Hemoglobin values were compared separately for female and male students in terms of the number and percentage of students with normal hemoglobin levels and those with anemia. Records of patients visiting the MCH were analyzed for hemoglobin levels and other conditions.

## Results

The pretest and post-test results are summarized in Table 2. The age of the student was recorded from birthday by the calendar to the nearest of the year (<6 months and >6 months). A small but significant difference in age was observed between female (mean age 13.08 years) and male (mean age 13.37 years) students. No significant difference was observed in the pre- and post-test scores of female and male students.

**Table 2:** Age of students and scores obtained (out of 20) in pre- and post-tests

	Female students (N = 72)			Male students (N = 81)			All students (N = 153)		
	Age (y)	Pretest	Post-test	Age (y)	Pretest	Post-test	Age (y)	Pretest	Post-test
Minimum	13	0	12	13	0	12	12	0	12
Maximum	14	6	20	15	6	20	15	6	20
Mean	13.08	2.76	15.53	13.37	3.2	15.81	13.24	2.99	15.68
SD	0.278	1.16	1.96	0.679	1.785	2.356	0.535	1.53	0.278
p-value				0.001	0.08	0.042			

Only 127 (83%) students had brought signed informed consent forms. The rest reportedly had either forgotten to get it signed or forgot the signed form at home. As parental consent was required for blood sample collection, samples were collected only from the students who had brought the signed forms. Tables 3, 4 show the blood hemoglobin values of female and male students. Hemoglobin levels of more than 13.5 g/dL in males and more than 12 g/dL in females were taken as normal [16]. Hemoglobin levels between 10 g/dL and the lower limit of normal for gender were considered mild anemia, between 8 and 10 g/dL were considered moderate anemia, and between 6.5 and <8 g/dL were considered severe anemia.

**Table 3:** Blood hemoglobin values (g/dL) of all students who had submitted the informed consent form signed by a parent

	Female students	Male students
Number of students	58	69
Minimum	8.8 g/dL	10.8 g/dL
Maximum	14.4 g/dL	15.5 g/dL
Mean	12.04 g/dL	14.03 g/dL
SD	1.53	0.86

**Table 4:** Categorization of students into those with normal levels of hemoglobin, mild anemia, and moderate anemia

	Normal Hb	Mild anemia	Moderate anemia
Female students (N = 58)	12-16 g/dL	10-11.9 g/dL	8-9.9 g/dL
Number (%) of students with condition	33 (56.9%)	19 (32.8%)	6 (10.3%)
Male Students (N = 69)	13.5-18 g/dL	10-13.4 g/dL	8-9.9 g/dL
Number (%) of students with condition	56 (81.16%)	13 (18.8%)	0

Thirty-three (56.9%) female and 56 (81.2%) male students had normal hemoglobin levels for their gender. Nineteen female and 13 male students had mild anemia, while six female students had moderate anemia. A total of 38 out of 127 (29.9%) students had anemia. All had microcytic hypochromic anemia, suggesting iron deficiency. Reports were provided to the students the next day. Eleven students who appeared thin were surprised to find they had normal levels of hemoglobin. Three students with overweight/obesity had mild/moderate anemia. Students requesting a repeat test were asked to visit the outpatient department (OPD) of the MCH. This showed the students that blood hemoglobin level was not affected by the weight of the person: People with obesity can also be anemic.

A total of 32 people visited the free Anemia Awareness Camp organized by the MCH on information received from the students. Table 5 presents the details of these patients.

**Table 5:** Details of persons visiting OPD of local district hospital on receiving information from students who had attended the Anemia Awareness Program at school OPD: Outpatient department.

	Female	Male
N	18	14
Age (years)	9-45	2-68
Normal Hb	2	2
Mild anemia	2	7
Moderate anemia	14	6
Minimum Hb	8.2 (g/dL)	8.2 (g/dL)
Maximum Hb	12.4 (g/dL)	14.2 (g/dL)

Two female and two male persons had normal hemoglobin levels. Two males and seven females had mild anemia, and 14 females and six males had moderate anemia. Detailed complaints and conditions are presented in Table 6.

**Table 6:** Detailed complaints and conditions of persons visiting the OPD according to age groups OPD: Outpatient department; Hb: Hemoglobin; F: Female; M: Male; T2DM: Type 2 diabetes mellitus; BP: Blood pressure; HT: Hypertension.

Age (Years)	Gender		Remarks
	Female	Male	
<10	3	4	Pica/lethargy/weakness; normal Hb: 2 (1F, 1M); moderate anemia: 4 (2 F, 2M)
10-20	7	3	Weakness/lethargy; normal Hb: 1 (F); mild anemia: 2 (M); moderate anemia: 7 (6F, 1M)
20-40	7	0	Weakness/lethargy; mild anemia: 2 (pregnant); moderate anemia: 5 (1 pregnant, 1 undiagnosed T2DM, 1 jaundice)
40-60	1	6	Weakness/lethargy/weight loss; normal Hb: 1 (M); mild anemia: 3 M (2 undiagnosed T2DM, 1 previously diagnosed diabetic on hypoglycemic oral medicine, anti-hypertensive medicine with high BP; 1 undiagnosed HT); moderate anemia: 3 (1 F with pre-diabetes, undiagnosed HT, low protein; 2 M, one previously diagnosed diabetic with undiagnosed HT)
>60	0	1	Weakness, moderate anemia, hypertensive on anti-HT medication

Three persons had undiagnosed type 2 diabetes mellitus, while three had undiagnosed hypertension. One 38-year-old female had undiagnosed regurgitation hyperbilirubinemia and was referred for ultrasonography of the abdomen. One four-year-old male child with pica and a blood hemoglobin level of 8.8 g/dL was referred for a lead test based on the occupational history of the father. Two males (58 years and 68 years) with moderate anemia reported occasional mild pain in the abdomen with loose stools and were advised stool tests.

## Discussion

Despite the economic progress made by the country in recent years and multiple nutritional programs started by the government, the nutritional status of children and women is still not satisfactory [17]. The government of India has taken up multiple initiatives to eradicate anemia. The National Nutritional Anemia Control Program (NNACP) which was started in 1970 was initiated to provide iron and folic acid (IFA) tablets to all vulnerable groups [18]. The Integrated Child Developmental Services (ICDS) program was initiated by the Indian Government to provide nutritional meals, primary healthcare, immunization, health check-ups, referral services, and preschool education to children under six years of age [19]. This program was discontinued in 1978 and restarted in the 10th Five-Year Plan (2002-2007). The Weekly Iron and Folic Acid Supplementation (WIFS) program with biannual helminthic control was recommended by the World Health Organization to control anemia in women of reproductive age. WIFS was started in 2012 by the Indian Government for 10- to 19-year-olds [20]. The Ministry of Health and Family Welfare launched the National Iron Plus Initiative (NIPI) in 2013 [21]. Anemia Mukta Bharat (AMB) under the POSHAN program was started in 2018 [22] and aims to reduce anemia in six beneficiary age groups: infants and toddlers (6-59 months), children (5-9 years), adolescents (10-19 years), pregnant and lactating women, and women of reproductive age group (15-49 years).

One hindrance to the progress of these programs is the lack of awareness in the general population [15]. Imparting health education to the affected persons can increase adherence to the health programs and reduce attrition rates. As door-to-door educational programs are not feasible, we targeted the most accessible population. Health literacy programs designed for school children are effective [23]. In this study, a middle school with students belonging to a rural background was selected. The school principal kindly agreed to our request to run this program. The students as well as faculty and non-teaching staff were eager to attend the program. As seating space was limited, only students of class eight were selected to attend the program as they would be leaving the school in a few months.

Results of pre- and post-tests (Table 2) revealed that the students gained knowledge from the program. A similar study was conducted in Delaware, where physicians discussed health topics with class seven students, which showed increased short-term knowledge [24].

Forty-three percent (25 out of 58) of girls had mild/moderate anemia, compared to 19% (13 out of 69) of boys (Tables 3, 4). Banerjee et al. [25] reported an anemia prevalence of 54.2% in 12-14-year-old girls and 45.8% in 12-14-year-old boys. Kuppusamy et al. [26] have reported an anemia prevalence of 61.5% in adolescent females. Our sample is much smaller and from a single standard of a single school.

Students with mild and moderate anemia were provided lists of iron-rich food items, hygiene tips, and handouts of the lectures. Snacks provided during the awareness program included locally available iron-rich items: black gram, sweets made of jaggery-peanut-amaranth-seed (rajgira-peanut chikki), and dates.

Thirty-two persons visited the Anemia Awareness Camp of the MCH at the suggestion of the school students (Table 5). This indicates that the students were enthusiastic about spreading their newly gained knowledge. Some of the persons visiting the OPD had other complaints besides anemia (Table 6). Our team identified three persons with undiagnosed type 2 diabetes mellitus, one with pre-diabetes, three with undiagnosed hypertension, and one with jaundice.

The post-test form had space for feedback from the students. All students (100%) found this program interesting, informational, and beneficial for themselves and the society. We have listed the doubts raised by the students, faculty, and non-teaching staff attending the program in Table 7.

**Table 7:** The doubts raised by the students, faculty, and non-teaching staff attending the program

Questions
Milk is considered a complete food, so how can it be a poor source of iron?
Why can’t calcium and iron supplements be taken together?
Why should iron supplements not be taken after a fiber-rich meal or tea?
Why does heavy metal toxicity cause anemia?
How can poor hygiene cause anemia?

Our team addressed all queries raised and encouraged all persons attending the Awareness Program to spread information to as many people as possible.

Correct answers to all questions were provided at the end of the post-test to ensure awareness in every student. Doubts and queries of the attendees were addressed. The students were encouraged to form teams to spread awareness and to be on the lookout to identify family members with symptoms of anemia.

This pilot study indicates that teaching school students about anemia and its eradication is a relatively low-cost method to generate awareness among the students who spread the information to their families and acquaintances. We promoted the belief that young students can be responsible and bring about change in their society. The students were convinced that their knowledge could benefit others, and this increased their interest in the topic. Our team was happy when several students expressed their interest in becoming physicians and healthcare workers in the future.

Limitations of the study

Our biggest limitation was the small sample size. This was a self-funded project. Funds for snacks, questionnaires, feedback forms, travel, etc. were contributed by the team, while tests were performed in the free camp organized by the Maharshi Vashishtha Autonomous State Medical College Hospital, Basti, India.

## Conclusions

Physician-conducted awareness programs organized in schools can impart useful information to students that can help them protect themselves from a specific disease, identify symptoms of the disease in themselves and others, and inform other persons how to protect themselves from the disease. In this pilot study related to anemia awareness, students with anemia were identified and advised regarding food and hygiene and were provided iron-folic acid tablets. Visits of patients to the free Anemia Awareness Camp showed that the students had spread information with sincerity. This pilot study illustrates the role of local schools and their students, the community, hospitals, and socially responsible physicians in the public health system.
